# Biosafety Issues in Patient Transport during COVID-19: A Case Study on the Portuguese Emergency Services

**DOI:** 10.3390/ijerph21010099

**Published:** 2024-01-16

**Authors:** Pierre Vandenberghe, Luis Manuel Ladeira, Margarida Gil, Ivo Cardoso, Fatima Rato, Jessica S. Hayes, Maire A. Connolly, Jean-Luc Gala

**Affiliations:** 1 Centre for Applied Molecular Technologies (CTMA), Institute for Clinical and Experimental Research (IREC), Université Catholique de Louvain, Tour Claude Bernard, Avenue Hippocrate, 54-55, bte B1.54.01, 1200 Bruxelles, Belgium; p.vandenberghe@uclouvain.be; 2 Instituto Nacional de Emergência Médica, Rua Almirante Barroso, 36, 1000-013 Lisboa, Portugal; luis.ladeira@inem.pt (L.M.L.); margarida.gil@inem.pt (M.G.); ivo.cardoso@inem.pt (I.C.); fatima.rato@inem.pt (F.R.); 3 School of Health Sciences, College of Medicine, Nursing and Health Sciences, University of Galway, H91 TK33 Galway, Ireland; jessica.hayes@universityofgalway.ie (J.S.H.); maire.connolly@universityofgalway.ie (M.A.C.)

**Keywords:** biosafety, pandemic, patient transport, emergency medical services (EMS), COVID-19, first responders

## Abstract

During the COVID-19 pandemic, first responders faced significant biosafety challenges, especially while handling patient transport, potentially exposing them to infection. The PANDEM-2 (European project on pandemic preparedness and response) project, funded by the Horizon 2020 program, sought to investigate the challenges confronting Emergency Medical Systems throughout the EU. First responders from Portugal’s National Institute of Medical Emergency (INEM) were considered as a representative operational model of the national first responder agencies of European member states because they played a critical role during the COVID-19 pandemic. As a result, they were asked to complete an online survey about their COVID-19 pandemic-related professional activities. The survey focused on their perspectives on current biosafety guidelines and their operational practices. It covered opinions on existing protocols, technical concerns during patient transport, and issues after the patients arrived at the hospital. The key findings revealed concerns about risk assessment, the inadequacy of guidelines, and disparities in equipment access. This survey emphasizes the importance of developing streamlined, adaptable biosafety protocols, better coordination between prehospital and in-hospital services, and the development of scalable, cost-effective biosafety solutions. Based on our findings, we propose improvements to national and European biosafety directives and advocate for streamlined adaptation during pandemics.

## 1. Introduction

The response to the COVID-19 pandemic highlighted the critical role of Emergency Medical Services (EMS) in public health, safety, and the overall healthcare system. Working in confined spaces such as ambulances [1], aircrafts [2], or patients’ homes, surrounded by concerned family members in many situations [3], first responders faced an increased risk of being infected with SARS-CoV-2. Infection routes included respiratory droplets, contact with contaminated surfaces, and certain medical interventions known to increase the risk of virus transmission, such as airway management [4], the suction of secretions, or cardio-pulmonary resuscitation [5,6]. Globally, the logistical and educational challenges posed by the pandemic prompted EMS agencies to devise solutions that maximized safety while allowing for rapid adaption to the evolving crisis [7]. However, preliminary research has revealed shortcomings in resource allocation [8], continuing education [9], protocol adherence, and decontamination practices [10].

As a result, current research efforts are focusing on the complexities of developing and implementing effective national and European cross-border pandemic strategies. PANDEM-2 (Pandemic Preparedness and Response, https://pandem-2.eu/ (accessed on 10 January 2024)) is a Horizon 2020 project that has developed tools and systems to improve the EU’s cross-border pandemic health emergency preparedness and response. The innovative features of the PANDEM-2 prototype system enabled comprehensive data collection, analysis, and visualization with the goal of supporting rapid and evidence-based decision making by public health authorities in European Union member states (EU-MS) during future pandemics. This system provides the opportunity to train personnel in preparing for future pandemic challenges, providing flexibility for further development by end-users and enhancing their ongoing response capabilities [11,12,13,14].

One of the objectives of PANDEM-2 was to examine the challenges encountered by EMS across the EU. Portugal’s Instituto Nacional de Emergência Médica (INEM, National Institute of Medical Emergency) was chosen as a representative operational model for the national first responder agencies within the EU-MS. Indeed, the INEM plays a critical role in overseeing the country’s EMS and has adopted a range of strategies to address the challenges posed by the COVID-19 pandemic. These strategies included handling the daily tasks of responding to approximately 3500 emergency cases and managing almost 4000 emergency calls each day. The INEM also provided pre-hospital medical assistance to COVID-19 patients based on severity and emergency criteria and implemented various biosafety measures. Moreover, the INEM supported the Ministry of Health and other health authorities in both the national and international response to the pandemic. The diverse and reactive activities undertaken by the INEM during this period underscore its multifaceted role and exemplify the challenges faced by EMS during the COVID-19 pandemic, highlighting existing gaps in pandemic preparedness and response strategies among EU-MS.

The PANDEM-2 survey launched by the INEM focused on biosafety guidelines and operational practices during the COVID-19 pandemic, gathering feedback on existing protocols, technical concerns during patient transport, and issues after patients arrived at the hospital. During the pandemic, despite pre-existing guidelines on PPE use [8,9,10], the INEM observed discrepancies in the application of PPE by EMS responders [8,9,10]. This study highlights the critical need for strict hygiene standards, appropriate personal protective equipment (PPE), Standard Operating Procedures (SOPs) [15], and specialized equipment [2,9,10]. Furthermore, the current survey looks at the INEM’s practical experience as an end-user during the pandemic, outlining key lessons, identifying gaps in preparedness, and focusing on the retention of newly acquired skills.

## 2. Materials and Methods

### 2.1. Consortium Network

This cross-sectional study is a component of the project PANDEM-2 (Pandemic Preparedness and Response; 883285; https://pandem-2.eu/ (accessed on 10 January 2024)).

The project was initiated in response to the H2020-SU-SEC-2018-2019-2020 call on “Demonstration of novel concepts for the management of pandemic crises”.

The consortium is made up of EU experts from various sectors, including health (with organizations such as the INEM and UCLouvain spearheading this research), security, defense, microbiology, communications, information technology, and crisis management. 

### 2.2. EMS in Portugal

In Portugal, the National Institute of Medical Emergency oversees emergency medical services throughout the country. The INEM has a wide range of medical emergency resources to address various extra-hospital urgencies and emergencies. These include the following:

(a) Medical emergency ambulances and motorbikes operated by pre-hospital emergency technicians, firefighters, and professionals from the Portuguese Red Cross
(b) Specialized services: To respond to the COVID-19 pandemic’s challenges, the INEM implemented special measures in March 2020. They launched a dedicated pre-hospital transport service specifically for patients suspected or confirmed to have SARS-CoV-2 infection. The teams had received adequate training in the use of PPE. This initiative ensured that the ambulances operated beyond the regular fleet and underwent specific disinfection procedures. Some ambulances were equipped with isolation cells, designated clean/dirty zones, and specialized waste management systems.

Furthermore, during the pandemic’s peak, a dedicated decontamination line was established in partnership with the National Guard (GNR). This streamlined process enhanced safety, increased the decontamination efficiency, and ensured ambulances were rapidly available for the next patient transfer missions.

### 2.3. Online Survey

A questionnaire was developed to assess biosafety guidelines for patient transport and management during the COVID-19 pandemic. Questions were created based on an in-depth analysis of relevant standard operating protocols and guidelines sourced from emergency medical institutions in various European countries. Technical questions were formulated based on identified commonalities in decontamination techniques, vehicles used for patient transport, PPE utilization, and training programs. Following that, the questionnaire was refined through a feedback process with first responders from the Italian Red Cross, Austrian Red Cross, and INEM, incorporating their practical insights and experiences. Finally, the questionnaire was distributed to healthcare professionals and biosafety specialists within the PANDEM2 consortium for validation. The final questionnaire contained 19 English-language questions organized into three distinct sections (Table 1):

(a) General Biosafety Guidelines: This section had four questions focusing on the availability, accessibility, understandability, and applicability of biosafety guidelines. Each question offered response options of YES, PARTIALLY, or NO, along with a request for a brief explanation.
(b) Patient Transport Procedures: Comprising five main YES/NO questions, this section delved into the vehicles used for patient transport, first responders’ training, personal protective equipment, and decontamination methods. It also included additional multiple-choice questions related to these topics.
(c) Hospital Arrival Protocols: This section presented four main YES/NO questions addressing biosafety procedures upon a patient’s arrival at the hospital. Topics included the use of secure routes, the configuration of hospital rooms, and decontamination methods.

The survey was hosted on Google Forms at the end of February 2022 (https://forms.gle/meiMaQhMaYegfovk6) and was available for 2 months. The survey link was distributed to first responders from INEM professionals, fire departments, and the Portuguese Red Cross via internal email by the heads of our consortium partner, INEM. Answers written in Portuguese were translated into English by INEM partners.

### 2.4. Legal Statement

All information provided was anonymized. Participants were given information and consent sheets prior to completing the survey. The legal bases for processing personal data are consent (GDPR Art. 6a), research purposes (GDPR Art. 6e), and administrative reasons (GDPR Art. 6f).

### 2.5. Statistics

To depict the numerical distribution of responses within each profession, descriptive statistics were used, and percentage representation was used for response patterns across all professions. Graphs were generated using Prism 9 software (Version 9.0.0 (121), GraphPad Software, Inc., La Jolla, CA, USA). The analysis of internal consistency was carried out using Cronbach’s alpha coefficient.

## 3. Results

### 3.1. Survey Answers Analysis

The following results provide insights from Portuguese stakeholders, positioning Portugal as a representative European nation. The questionnaire was completed by a total of 108 EMS stakeholders, including ambulance technicians (*n* = 46), firefighters (*n* = 33), nurses (*n* = 22), and physicians (*n* = 7). The measured Cronbach’s alpha coefficient of 0.81 indicated that the survey had good internal consistency.

Table 2 displays the number of responses obtained in each profession for the questions in the three sections of the survey, along with the overall response rate across all professions.

#### 3.1.1. First Section: Biosafety Guidelines

- Adherence and Accessibility: Nearly all (98%) first responders adhered to biosafety guidelines for patient transport. Furthermore, about 82% found these guidelines accessible and comprehensible.
- Sources of Guidelines: The investigation revealed that these guidelines, primarily at the national/local level, were disseminated mostly through email or internal communication channels.
- Challenges: Notably, up to 30% of first responders stated difficulties in adhering to biosafety guidelines during a crisis such as the COVID-19 pandemic. Specifically, ambulance technicians and firefighters expressed the highest levels of misunderstanding and challenges in compliance.
- Feedback: Negative feedback was collated, revealing three primary concerns:
  - Frequent changes to biosafety protocols during the COVID-19 pandemic made them challenging to keep up with.
  - A perceived overemphasis on caution at the expense of effective risk assessment.
  - Some protocols seemed ill suited for real-world “street emergencies”.

Conclusion of the first section: This section highlights the main challenges first responders faced: the frequent alterations to biosafety protocols and an apparent lack of robust risk assessment.

#### 3.1.2. Second Section: Vehicles, Personal Protective Equipment, and Training

- Vehicles:
  - Only 34% of these ambulances were equipped specifically for COVID-19 positive patients, featuring isolation cells, HEPA filters, or ventilation systems.
  - Those without specialized vehicles attempted to minimize disposable item usage and equipment exposure.
  - Overall, 62% decontaminated their vehicles in dedicated locations with clear divisions between clean and contaminated zones, along with waste management facilities.
- PPE and Training:
  - Overall, 80% of first responders received training on PPE usage, with consistent training rates across professions.
  - In the pandemic’s early days (February–March 2020), 78% underwent “Just-in-time” training, covering PPE use, transportation, and decontamination. This combined online resources with hands-on PPE practice.
  - Concerningly, 38% lacked a specialized area for PPE removal, which ideally would separate clean and dirty zones and offer staff assistance for safe PPE removal and waste handling.

Conclusion of the second section: This section’s primary concerns revolved around insufficient vehicle adaptations for COVID-19 patients and the absence of dedicated areas for PPE use and vehicle decontamination.

#### 3.1.3. Third Section: Managing Patients in the Hospital

  - A total of 59% of first responders worked with a dedicated COVID-19 management center.

Transfer Protocols:

  - Overall, 72% used PPE on patients during transfers to minimize contact between patients and hospital staff or visitors.
  - Upon arrival, 58% of COVID-19 positive patients were placed in high-level isolation rooms (HIRs)—specialized negative-pressure spaces with frequent air changes and an anteroom.

Conclusion of the third section: Despite rigorous regulations, many first responders lacked the infrastructure needed to manage COVID-19 positive patients safely. Furthermore, in almost one out of every five cases, PPE was not used for patients during transfer.

Table 3 displays the percentage of selected responses among initial respondents regarding the equipment and methods used during patient transport to the hospital.

- Ambulances were the primary mode of patient transportation across professions.
- Hand cleaning by employees was the primary decontamination method, though fogging and air filtration were also employed, especially by firefighters.
- Standard PPE included gloves, FFP2/FFP3 masks, and body coveralls, with many also using goggles/face shields and footwear protection. Surgical masks were rarely used.
- Most room decontamination was carried out through hand cleaning, but less than 50% incorporated air/fogging and UV decontamination methods.

## 4. Discussion

Our study highlighted the specific challenges faced by first responders during the COVID-19 pandemic, particularly in adhering to existing and especially new biosafety guidelines. The constant flow of new SARS-CoV-2 medical information (such as transmission modes, the R0 reproduction number, and evolving SARS-CoV-2 virulence) combined with Europe’s PPE shortage [16,17] made risk assessment more difficult for biosafety experts [18]. As a result, inappropriate biosafety guidelines or outdated guidelines may exacerbate EMS staff fatigue and stress [19], induce over-protection or under-protection against the biological threat [20], and reduce trust in biosafety advisories [21]. Our findings also suggest that healthcare workers who have only received “basic life support” training may struggle with complex biosafety terminology. These terms should be included in training via e-learning, simulations, videos, and live demonstrations to bridge this gap, as these methods have been shown to be effective for less-trained EMS personnel [22]. Given the widespread use of smartphones among stakeholders [23,24], it is critical that biosafety information is quickly accessible to healthcare professionals during the acute phase of a pandemic. Visually appealing flyer formats that can incorporate QR codes could be an effective tool.

It is critical for biosafety management to improve coordination between pre-hospital and in-hospital care services. While national efforts, led by entities such as INEM, aimed to foster safe work environments as COVID-19 emerged, rapid EMS overloads resulted in disparities in biosafety provisions among first responders. This highlights the need to identify and develop scalable, cost-effective biosafety solutions. These measures, whether personal protective equipment, decontamination methods, or hospital configurations, should be available to all EMS tiers and adaptable to any hospital’s infrastructure. Technically, improving pandemic preparedness and the responses of first responders requires the development and availability of standardized equipment, vehicle modifications, and decontamination techniques that protect stakeholders regardless of pathogen characteristics. Notably, our survey confirmed that manual hand cleaning was the most commonly used decontamination method. While it is less expensive, it takes more time and poses contamination risks to emergency personnel [25,26]. “No-contact” techniques such as chlorine dioxide or hydrogen peroxide vaporization and UV-light decontamination [27] have been available for many years. Recent efforts have increased their effectiveness, affordability, and inclusion in ambulance decontamination protocols [28,29].

The primary limitation of this study is the potential for response redundancy in technical questions. Respondents having similar jobs and working in the same department are likely to use the same transport vehicles, undergo similar training, use identical PPE, and follow decontamination protocols. This similarity could limit the diversity of perspectives and experiences in the dataset. Conversely, it is plausible that workers in the same department perceive risks and shortcomings similarly. Another challenge is the reliance on participants’ self-adherence, making it difficult to control the participation rate, which affects the study’s power. While the INEM serves as a valuable EU case study, it is crucial to expand the survey to other EU-MS first responder organizations. Before we do so, we need to consider other potential limitations of such studies, such as time constraints, misunderstanding of the subject matter, and, notably, language barriers. These issues may cause healthcare providers to be hesitant or unable to complete the survey. Understanding the various backgrounds of our target audience is critical for broader outreach. This involves tailoring our survey style to accommodate language diversity, emphasizing the importance of the survey, and leveraging incentives such as endorsements from direct hierarchies and crafting country-specific communication strategies.

## 5. Conclusions

This study highlighted the significant disruptions that large-scale pandemics, such as the COVID-19 pandemic, can inflict on what are believed to be well-established first-responder healthcare protocols. Challenges such as inadequate risk assessment, unequal access to personal protective equipment (PPE), and a lack of biosafety training specifically tailored for pandemic conditions have jeopardized first responders’ safety and well-being. Protecting these stakeholders in the healthcare system is critical, given their roles in managing, transporting, hospitalizing, and treating patients either suspected or confirmed to be infected. The findings of the study highlight the need for the development of a generic epidemic–pandemic toolkit designed to provide first responders with quick, easy, and continuous access to current and continuously updated relevant information and very practical biosafety recommendations. The creation of this toolkit aims to offer implementable solutions at both national and European levels, thereby enhancing preparedness and response capabilities for future pandemics.

## Figures and Tables

**Table 1:** Detailed questions from the three sections of the survey.

Section/Question	Description/Options
**1. Biosafety Guidelines**	*Definition: Biosafety refers to “the containment principles, technologies and practices that are implemented to prevent unintentional exposure to pathogens and toxins, or their accidental release” (WHO)*
1.1	Do your institution follow biosafety guidelines for patient transport to hospital? *- [ ] YES—[ ] NO*
1.2	If Yes, from where did you get/follow biosafety guidelines? *- [ ] WHO—[ ] CDC—[ ] ECDC—[ ] National guidelines—[ ] Local guidelines—[ ] Other:* ________
1.3	Are the biosafety guidelines you are following easily available and accessible? *- [ ] YES—[ ] PARTIALLY—[ ] NO. Provide a short explanation for your answer:* ________
1.4	Are the biosafety guidelines you are following comprehensive? *- [ ] YES—[ ] PARTIALLY—[ ] NO Provide a short explanation for your answer: ________*
1.5	Are those biosafety guidelines easy to follow during crisis such as COVID-19 pandemic? *- [ ] YES—[ ] PARTIALLY—[ ] NO Provide a short explanation for your answer: ________*
**2. Vehicles, personal protective equipment and training**	*In terms of COVID-19 pandemic timeline, we are asking you to answer when you were at a later stage of the pandemic.*
2.1	Which patient’s transport vehicles do you use? *- [ ] Ambulance—[ ] Plane—[ ] Helicopter—[ ] Train—[ ] Other: ________*
2.2	Do you have vehicles designed and adapted for COVID-19 patient transport? *- [ ] YES—[ ] NO If Yes, which modifications have been applied? ________*
2.3	Which Personal Protective Equipment do you use? *- [ ] Gloves—[ ] Googles/Face shield—[ ] Mouth mask (FFP2/FFP3)—[ ] Footwear protection—[ ] Body coverall—[ ] Other: ________*
2.4	Do you have a specific place for PPE removal procedures? *- [ ] YES—[ ] NO*
2.5	Which vehicle decontamination method do you use? *- [ ] Hand cleaning by staff—[ ] Fogging—[ ] UV Light—[ ] Air filtration—[ ] Other: ________*
2.6	Do you have a specific place for vehicle decontamination? *- [ ] YES—[ ] NO*
2.7	Is your staff trained for PPE donning/doffing procedures? *- [ ] YES—[ ] NO*
2.8	Did your staff have a “just-in-time” biosafety training for COVID-19 pandemic? *- [ ] YES—[ ] NO* *If Yes, could you specify when this just-in-time training was introduced and what was the subject (ex: PPE donning/doffing, decontamination procedure...)? ________*
2.9	Which support are you using to provide training material to your staff? ________
**3. Patient handling at the hospital**	
3.1	Do you have a specific COVID-19 management center? *- [ ] YES—[ ] NO*
3.2	Upon arrival, do you have a secured route for patient transfer from vehicle to hospital bed? *- [ ] YES—[ ] NO*
3.3	Are PPE used for patients also? *- [ ] YES—[ ] NO*
3.4	Are all confirmed COVID-19 positive patients placed in high level isolation rooms? *- [ ] YES—[ ] NO*
3.5	Which room decontamination method do you use? *- [ ] Hand cleaning by staff—[ ] Fogging—[ ] UV Light—[ ] Air filtration—[ ] Other: ________*

**Table 2:** Detailed number of responses obtained for the main questions of the whole survey.

	Number of Responses Per Profession					
Questions	Responses	Ambulance Tech.	Firefighters	Nurse	Physician	Total
**1. Biosafety Guidelines**						
Does your institution follow biosafety guidelines for patient transport to hospital?	YES	45	32	22	7	106 (98%)
	PART.	0	0	0	0	0 (0%)
	NO	1	1	0	0	2 (2%)
Are the biosafety guidelines you are following easily available and accessible?	YES	32	30	21	6	89 (82%)
	PART.	12	2	1	1	16 (15%)
	NO	2	1	0	0	3 (3%)
Are the biosafety guidelines you are following comprehensive?	YES	33	29	21	5	88 (81%)
	PART.	11	3	1	2	17 (16%)
	NO	2	1	0	0	3 (3%)
Are those biosafety guidelines easy to follow during crisis such as COVID-19 pandemic?	YES	25	28	17	3	73 (68%)
	PART.	18	4	5	3	30 (28%)
	NO	3	0	0	1	5 (5%)
**2. Vehicles, personal protective equipment, and training**						
Do you have vehicles designed and adapted for COVID-19 patient transport?	YES	9	18	8	1	36 (34%)
	NO	36	15	14	6	71 (66%)
Do you have a specific place for PPE removal procedures?	YES	27	23	12	4	66 (62%)
	NO	18	20	10	3	41 (38%)
Do you have a specific place for vehicle decontamination?	YES	24	21	18	3	66 (62%)
	NO	21	12	4	4	41 (38%)
Is your staff trained for PPE donning/doffing procedures?	YES	30	28	21	5	84 (79%)
	NO	14	5	1	2	22 (21%)
Did your staff have a “just-in-time” biosafety training for COVID-19 pandemic?	YES	32	28	19	4	83 (78%)
	NO	13	5	3	3	24 (22%)
**3. Patient handling at the hospital**						
Do you have a specific COVID-19 management center?	YES	26	19	15	3	63 (59%)
	NO	19	13	7	4	43 (41%)
Upon arrival, do you have a secured route for patient transfer from vehicle to hospital bed?	YES	25	24	19	7	75 (72%)
	NO	19	7	3	0	29 (28%)
Are PPE used for patients also?	YES	29	23	19	4	75 (72%)
	NO	15	8	3	3	29 (28%)
Are all confirmed COVID-19 positive patients placed in high level isolation rooms?	YES	22	21	13	2	58 (58%)
	NO	18	10	9	5	42 (42%)

**Table 3:** Equipment and methods used among stakeholders.

	Rate of Chosen Responses among All First Responders (%)	
Equipment and Methods	Responses	Total
Patient transport vehicles	Ambulance	100
	Helicopter	13
	Plane	0
	Train	0
	Other	3
Personal Protective Equipment	Gloves	94
	Goggles/Face Shield	86
	Mouth mask (FFP2/FFP3)	98
	Footwear protection	83
	Body coverall	95
Vehicle decontamination method	Hand Cleaning	96
	UV Light	5
	Fogging	22
	Air Filtration	13
Patient room decontamination method	Hand Cleaning	94
	UV Light	7
	Fogging	24
	Air Filtration	17

## Data Availability

The data presented in this study are available upon request from the corresponding author. The data are not publicly available out of respect to the privacy of the study participants.
